# Tumor-derived IL-18 induces PD-1 expression on immunosuppressive NK cells in triple-negative breast cancer

**DOI:** 10.18632/oncotarget.16281

**Published:** 2017-03-16

**Authors:** In Hae Park, Han Na Yang, Kyoung Joo Lee, Tae-Sik Kim, Eun Sook Lee, So-Youn Jung, Youngmee Kwon, Sun-Young Kong

**Affiliations:** ^1^ Center for Breast Cancer, National Cancer Center, Korea; ^2^ Breast and Endocrine Cancer Branch of Research Institute, National Cancer Center, Korea; ^3^ Department of System Cancer Science, Graduate School of Cancer Science and Policy, National Cancer Center, Korea; ^4^ Translational Epidemiology Research Branch, Research Institute, National Cancer Center, Korea; ^5^ Department of Laboratory Medicine, Hospital, National Cancer Center, Korea

**Keywords:** IL-18, NK cells, breast cancer, hormone receptor, PD-1

## Abstract

**Purpose:**

While the inflammatory cytokine interleukin-18 (IL-18) is known to activate natural killer (NK) cells, its precise role in cancer is controversial. In this study, we investigated the role of tumor-derived IL-18 on peripheral blood NK cells in breast cancer patients.

**Results:**

In breast cancer cell lines, IL-18 was expressed and secreted in the triple-negative breast cancer (TNBC) cell lines MDA-MB-231 and HCC-70 but not in MCF-7 cells. The immature and non-cytotoxic CD56^dim^CD16^dim/−^ NK cell fraction was increased following co-culture with MDA-MB-231 cells, and this increase was not observed with tumor cells transfected with siRNA for IL-18 or in MCF-7 cells. In addition, tumor-derived IL-18 increased PD-1 expression on CD56^dim^CD16^dim/−^ NK cells, although no effect on PD-L1 expression in tumor cells was observed. Among EBC patients, serum IL-18 levels were significantly increased in those with a TNBC subtype compared to levels from patients with other subtypes, and the IL-18 levels were strongly associated with poor survival. Similarly, serum IL-18 and CD56^dim^CD16^dim/−^ NK cells were also increased in patients with metastatic TNBC who had progressive disease following cytotoxic chemotherapy.

**Experimental Design:**

We performed *in vitro* experiments in breast cancer cell lines, measured cytokine levels by RT-qPCR, western blot, and ELISA, and analyzed NK cell subsets by flow cytometry. For clinical validation, we collected and analyzed blood sample from patients with early breast cancer (EBC, *N* = 545) and metastatic breast cancer (MBC, *N* = 42).

**Conclusions:**

Our data revealed that tumor-derived IL-18 is associated with bad prognosis in patients with TNBC. Tumor-derived IL-18 increased the immunosuppressive CD56^dim^CD16^dim/−^ NK cell fraction and induced PD-1 expression on these NK cells.

## INTRODUCTION

Natural killer (NK) cells are a type of cytotoxic lymphocyte critical to the innate immune system [1]. NK cells recognize target cells in the absence of antibodies and MHC class I “self-markers,” provoking much faster immune reactions unlike other immune cells [2]. In addition, NK cells are capable of lysing antibody-coated target cells via a process known as antibody-dependent cellular cytotoxicity (ADCC). Currently, one of most important cancer therapies involves targeted treatment with monoclonal antibodies, such as trastuzumab and pertuzumab for breast cancer, cetuximab for colorectal cancer, and others. From this point of view, NK cells are expected to play a major role in cancer therapy during the effector phase of the adaptive immune response [1, 2]; however, so far, relatively few studies have investigated the clinical implications of NK cells, especially in breast cancer.

NK cells are activated in response to many cytokines, including interleukin (IL)-12, IL-15, IL-18, IL-2, and CCL5 [3]. Among those, IL-18 plays both pro-inflammatory and anti-cancer roles in cancer progression [4]. IL-18 is expressed and secreted by immune cells and directly activates NK cells by regulating interferon (IFN)-γ promoter activity [5, 6]; however, in some cancers, such as melanoma and pancreatic cancer, IL-18 is secreted, and high levels of serum IL-18 are correlated with poor prognosis in multiple cancer types [7, 8]. While the mechanism of action remains obscure, many studies have shown that tumor derived IL-18 not only provokes immuno-ablative NK cell expansion (i.e., immunosuppressive subsets) but also drives premature NK cell death [9, 10].

Previously, the role of tumor derived IL-18 has not been investigated in terms of the effects on breast cancer and NK cells. In this study, we investigated the role of tumor derived IL-18 and its effects on NK cells. We also examined the association between serum IL-18 levels and patient prognosis with respect to survival and response to chemotherapies. This study was supported by NCC Grant No 1310340.

## RESULTS

### IL-18 production by breast cancer cell lines

To determine whether breast cancer cell lines (MCF-7, T47D, SKBR3, JIMT-1, MDA-MB-231, and HCC-70) express and secrete IL-18, the levels of mature IL-18 (18 kDa) were measured in cell lysates by western blot, and gene expression was quantified by RT-qPCR (Supplementary Figure 1A and 1B). IL-18 expression was higher in the triple negative breast cancer (TNBC) cells (MDA-MB-231 and HCC-70) compared to that in the other subtypes. IL-18 was also detected in MDA-MB-231 cell culture supernatants by ELISA assay (Supplementary Figure 1C). Subsequent transfection of MDA-MB-231 cells with three different IL-18 siRNAs significantly suppressed IL-18 production compared to transfection with control siRNA (Supplementary Figure 2). We confirmed that the transfection efficiency was highest 72 h after treatment. Therefore, subsequent experiments were carried out using tumor cells treated with siRNA for 72 h.

### Altered proportions of NK cell subpopulations following co-culture with IL-18-secreting tumor cells

**Figure 1:**
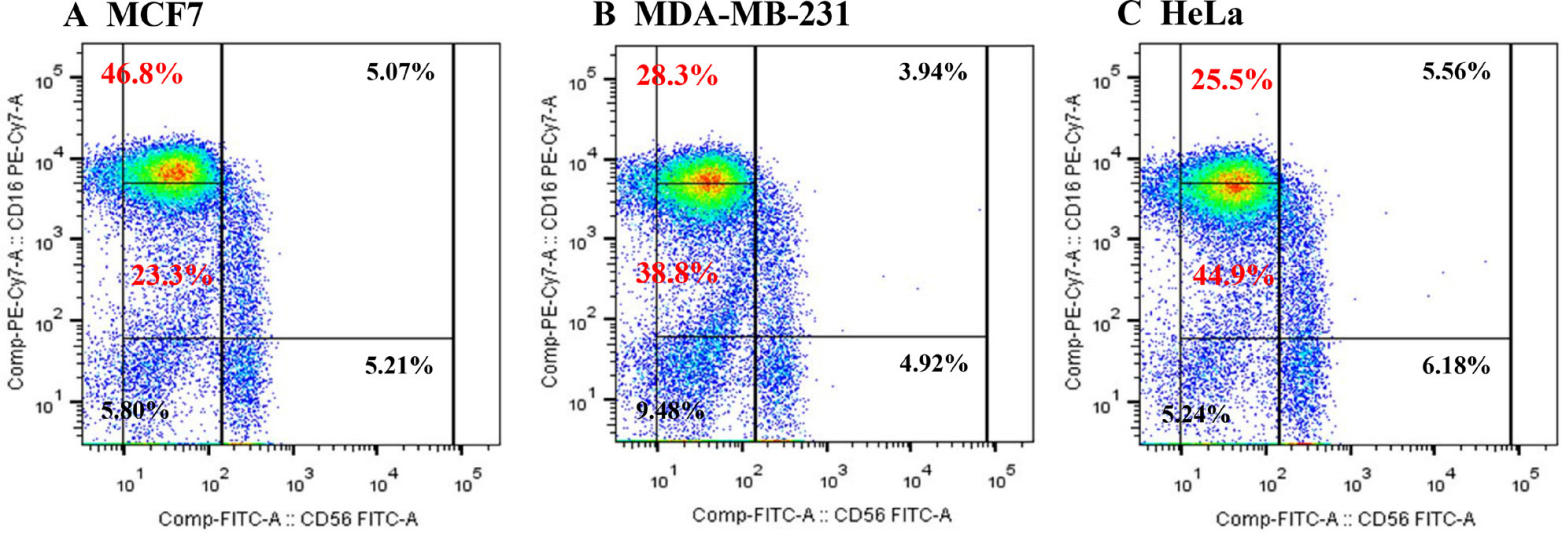
Proportions of NK cell subsets analyzed by flow cytometry after co-culture with MCF7 (**A**), MDA-MB-231 (**B**), or HeLa (**C**) cells.

**Figure 2:**
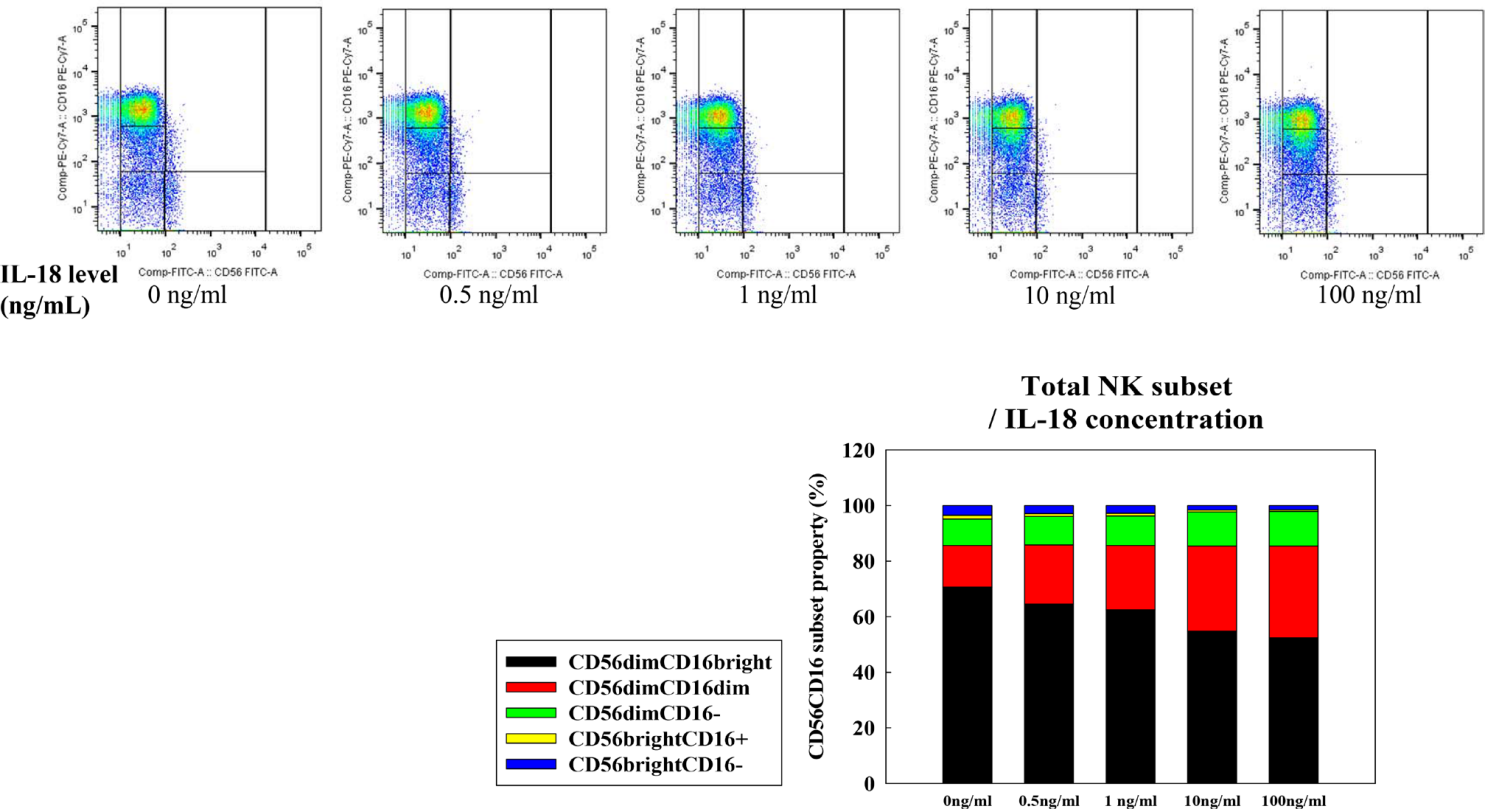
NK cell subset populations assessed following exposure to recombinant IL-18 NK cells were co-cultured with MCF-7 cells for 24 h prior to analyses of expression of the cell surface markers CD56 and CD16. The CD56^dim^CD16^dim/−^ NK cell subset was expanded in a dose-dependent fashion following exposure to IL-18.

We selected MCF-7 cells as a negative control and HeLa cells as a positive control for IL-18 production. Human NK cell subsets were analyzed for their expression of the CD56 and CD16 surface markers after co-culture with either MCF-7, MDA-MB-231, or HeLa cells for 72 h. The expression of CD16 on NK cells was significantly decreased following co-culture with MDA-MB-231 and HeLa cells compared to expression after co-culture with MCF-7 cells (28.3%, 25.5%, and 46.8%, respectively, Figure 1). Upon challenge of human NK cells with recombinant IL-18 in the MCF-7 co-culture system, the proportion of CD56^dim^CD16^dim^ NK cells increased in a dose-dependent manner (Figure 2).

**Figure 3:**
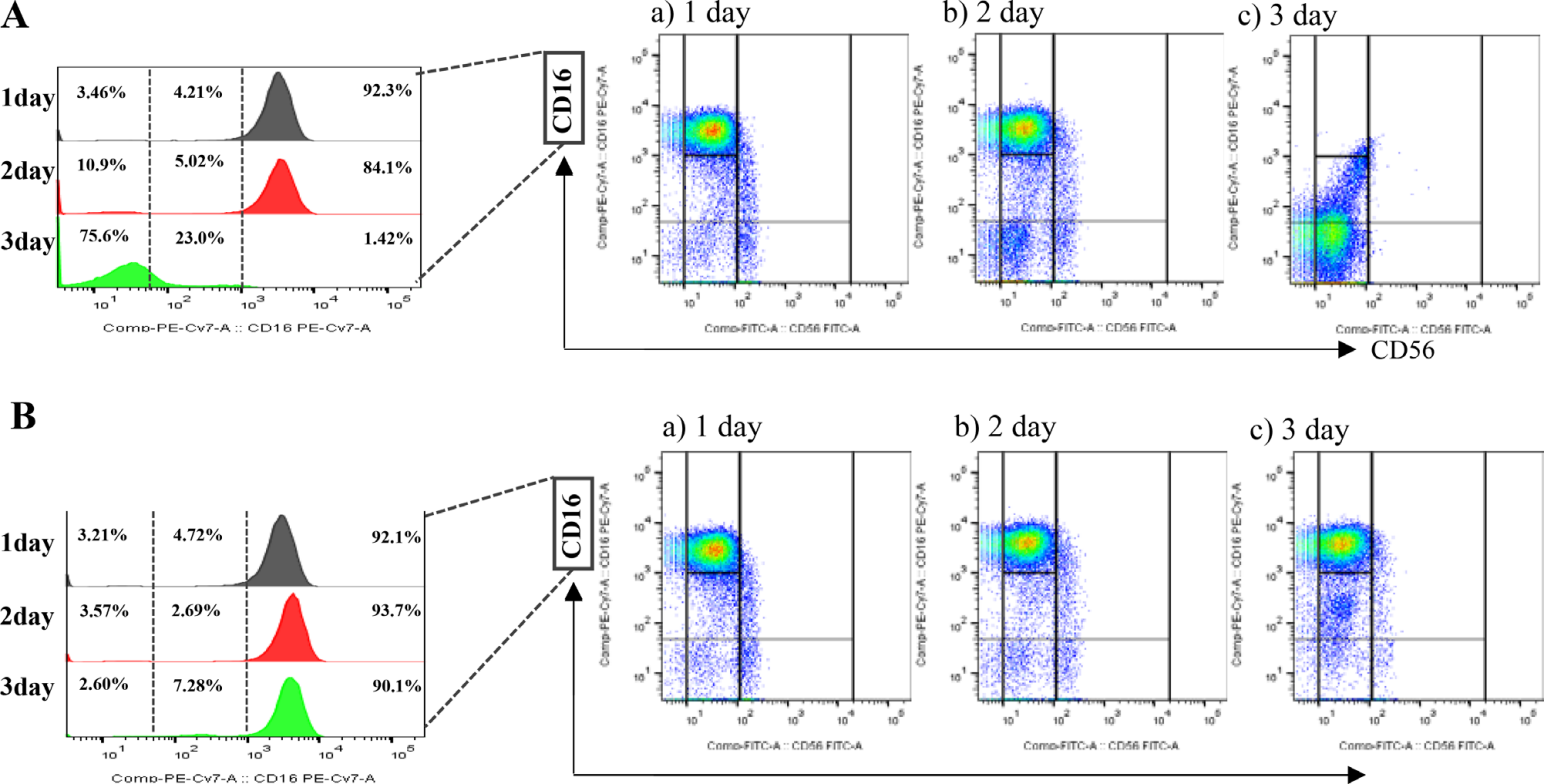
The effects of tumor-derived IL-18 on NK cell fractions Co-culture with MDA-MB-231*^siNC^* cells increased the proportion of CD56^dim^CD16^−^ NK cells (**A**). This increase was attenuated upon co-culture with MDA-MB-231*^siIL-18^* cells (**B**).

To verify the contribution of tumor derived IL-18 to the differentiation of NK cells, we depleted IL-18 using siRNA. When normal human NK cells were incubated with MDA-MB-231si-NC, the CD56^dim^CD16^−/dim^ NK subset increased (Figure 3A). Interestingly, this increase was attenuated when NK cells were cultured with IL-18 siRNA-transfected MDA-MB-231 cells (MDA-MB-231si-IL18) (Figure 3B). In contrast, co-culture with siRNA-transfected MCF-7 cells (both MCF-7si-NC and MCF-7si-IL18) failed to alter the NK cell subset populations (Supplementary Figure 3).

### Tumor-derived IL-18 enhances PD-1 expression on NK cells

**Figure 4:**
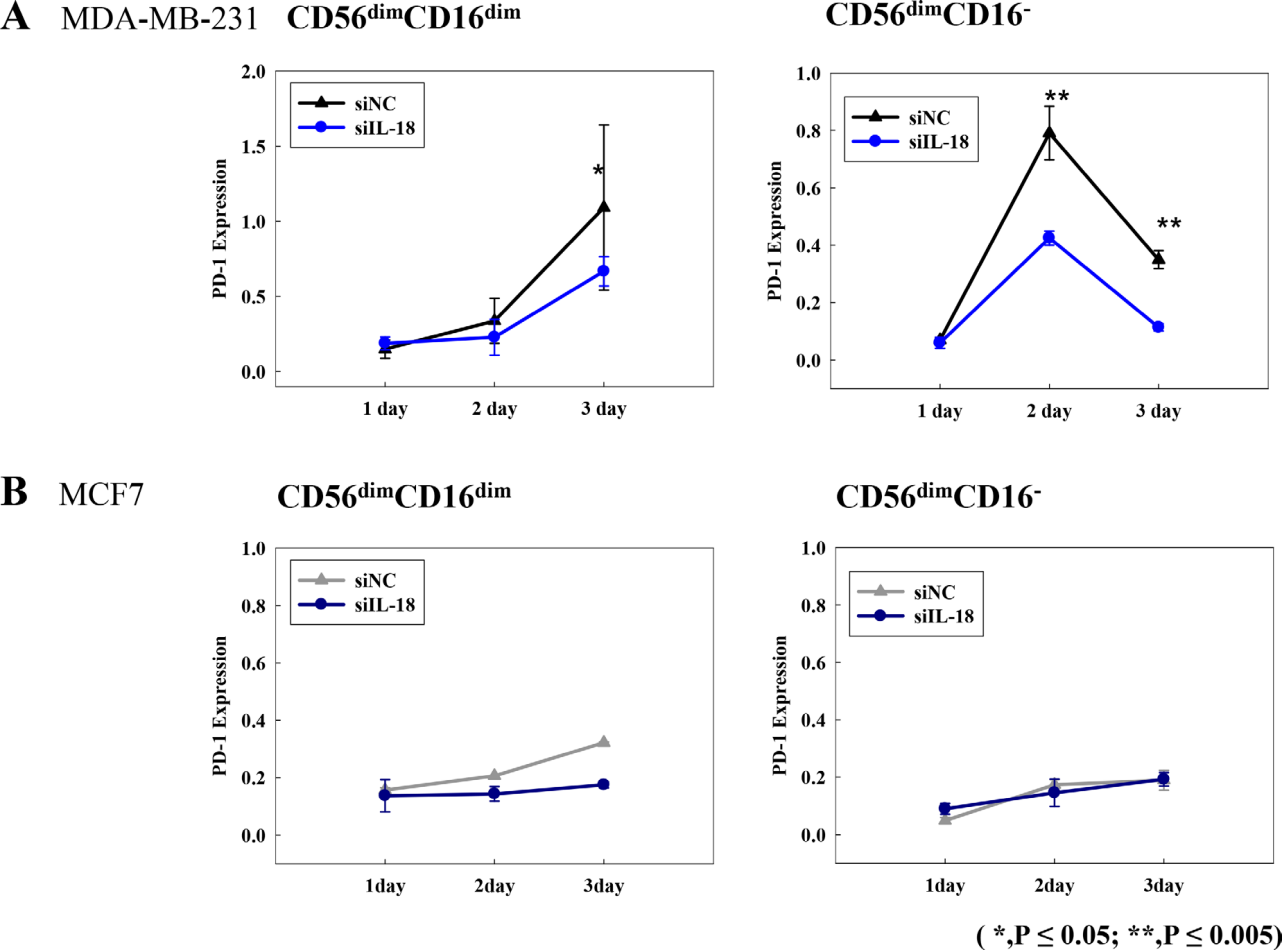
PD-1 expression on CD56^dim^CD16^dim/−^ NK cell subsets following co-culture with breast cancer cell lines MDA-MB-231*^siNC^* or MDA-MB-231*^siIL-18^* cells (A) and MCF-7*^siNC^* or MCF-7*^siIL-18^* cells (B) *p < 0.05; **p < 0.005. PD-1 expression was analyzed by flow cytometry. X-axis indicates the number of days following transfection.

We then investigated the immunosuppressive properties of CD56^dim^CD16^dim/−^ NK cells in terms of their expression of PD-1 using flow cytometry analysis. The increase of PD-1 expression was observed in immunosuppressive NK subsets (CD56^dim^CD16^dim/−^ NK cells subsets Figure 4A) co-cultured with MDA-MB-231*^siNC^* cells; however, incubation with MDA-MB-231*^si-IL18^* cells resulted in a significant attenuation of this effect (Figure 4A). Meanwhile, PD-1 expression was unchanged or decreased on CD56^bright^CD16^+^ NK cells or CD56^dim^CD16^bright^ NK cells, irrespective of the neutralization of IL-18 (Supplementary Figure 4A). Minimal expression of 107a and IFN-γ was detected in CD56^dim^CD16^dim/−^ NK cells subsets, and this expression was not changed by blocking tumor-derived IL-18 (Supplementary Figure 4B and 4C). PD-1 expression was not changed in CD56^dim^CD16^dim/−^ NK cells in co-culture with MCF7 cells regardless of blocking of IL-18 (Figure 4B). We also examined the effects of IL-18 on the expression of PD-L1 on tumor cells. PD-L1 expression on MDA-MB-231 cells was increased upon co-culture with human normal NK cells; however, depletion of IL-18 did not have any effect on PD-LI expression levels (Supplementary Figure 5).

### Serum IL-18 levels and survival of early breast cancer (EBC) patients

Next, we investigated the clinical implications of tumor derived IL-18 in EBC patients with respect to relapse and survival. Of a total of 545 EBC patients, the mean value of serum IL-18 was 352.9 ± 12.6 pg/mL. We also analyzed serum IL-18 levels according to hormone receptor (HR) and HER2 receptor status (HR+/HER2-, HR+/HER2+, HR-/HER2+, and HR-/HER2- subtypes). In agreement with previous cell line results, the serum levels of IL-18 were highest in patients with TNBC (HR-/HER2) and the lowest in patients with HR+/HER2- tumors among the four groups (HR+/HER2- [*N* = 228], 284.2 ± 18.4 pg/mL; HR+/HER2+ [*N* = 64], 314.1 ±33.3 pg/mL; HR-/HER2+ [*N* = 63], 313.8 ± 33.7 pg/mL; HR-/HER2- [*N* = 156], 444.3 ±23.4 pg/mL). In order to evaluate the association between serum IL-18 levels and clinical factors, we categorized patients into two groups according to their serum IL-18 levels, using 352.9 pg/mL as the cut-off value. As shown in Table 1, high serum IL-18 levels were significantly associated with poor prognostic factors, such as hormone receptor negativity (*p* < 0.001), larger tumor size (*p* = 0.005), nodal involvement (*p* = 0.021), and a higher Ki67 positivity (*p* = 0.013). High serum IL-18 levels were also correlated with shorter recurrence-free survival (RFS) and overall survival (OS), except in patients with HR+/HER2- tumors (Supplementary Figure 6A and 6B). Serum IL-18 levels remained as an important prognostic factor for both RFS and OS even after adjustment for other prognostic clinical variables, such as hormone receptor status, HER2 overexpression, tumor size, and nodal status (Supplementary Table 1).

**Table 1:** Patient characteristics (*N* = 545)

	High serum IL-18 (*N* = 200)	Low serum IL-18 (*N* = 345)	*p*-value
Age (median, range)	48.5 (27–85)	45 (24–78)	0.004
Hormone receptor (ER or PgR)			< 0.001
Positive	69 (34.5%)	223 (64.8%)	
Negative	131 (65.5%)	121 (35.2%)	
Not known	0	0	
HER2 receptor			0.032
Amplified	34 (17.0%)	93 (27.0%)	
Normal	144 (72.0%)	240 (69.6%)	
Not known	22 (11.0%)	12 (3.5%)	
Tumor size			0.005
≤5 cm	181 (91.0%)	332 (64.7%)	
>5 cm	18 (9.0%)	11 (3.2%)	
Nodal involvement			0.021
< 2	167 (83.5%)	311 (90.4%)	
≥ 2	33 (16.5%)	33 (9.6%)	
Ki67			0.013
< 14%	88 (44.0%)	197 (57.3%)	
14–20%	35 (17.5%)	48 (14%)	
≥ 20%	76 (38%)	99 (28.8%)	
Not known	2 (1.0%)	0	

IL, interleukin; ER, estrogen receptor; PgR, progesterone receptor; HER2, human epidermal growth factor receptor 2.

### Associations between higher serum IL-18 levels and immature NK cells and responses to cytotoxic chemotherapies in metastatic breast cancer (MBC) patients

Analyses were then performed for serum IL-18 levels and response to cytotoxic chemotherapy for patients with advanced MBC. Of 42 triple negative MBC patients, 19 showed a partial response (PR), and 23 patients exhibited progressive disease (PD) after 6 weeks of cytotoxic chemotherapy (Supplementary Table 2). We analyzed the serum IL-18 levels and the proportions of NK cell subsets using markers for CD56 and CD16 for these patients and compared the data to that from 24 healthy controls. We found that the IL-18 serum levels were higher in the patient groups compared to levels in the healthy controls (232.3 ± 43.2 ng/ml vs. 109.1 ± 6.2 ng/ml, *p* < 0.05). Serum IL-18 levels were also higher in patients with PD (276.0 ± 50.0 ng/ml) compared to PR (179.5 ± 28.8 ng/ml). In terms of NK subset analyses, CD56^dim^CD16^dim^ NK cells were most frequently observed in patients with PD (Supplementary Figure 7). Meanwhile, cytotoxic and mature NK cells (CD56^dim^CD16^bright^) or CD56^bright^CD16^+/−^ NK cell populations were significantly lower in patients with PD (Supplementary Figure 7) than in those with PR.

## DISCUSSION

Although the regulation of T cells has been widely investigated, the activity of the innate immune system in the context of cancer treatment has been overlooked. NK cells have a role in immune responses to tumor cells [13]. Many studies have shown that a specific NK cell subset may act as a negative regulator of the immune response and that activities of these immuno-ablative NK cell populations are dependent on the tumor microenvironment [9, 14]. IL-18 plays a critical role in inflammatory and immune responses, such as stimulation of NK and T cells and enhancement of Th1 responses [5, 11]. In line with this notion, IL-18 is considered to be a promising anti-tumor immunotherapy, either as a monotherapy or in combination with a monoclonal antibody [12]. Higher expression or secretion of IL-18, however, has been detected in various cancer cells, and tumor derived IL-18 has been shown to be a poor prognostic factor in cancer [11].

To more fully investigate the role of IL-18 in breast cancer, we examined the expression of IL-18 in various breast cancer cell lines and its effects on NK cells. Among the cell lines tested, IL-18 expression was significantly higher in cell lines derived from TNBC. Exposure to recombinant IL-18 or co-culture with IL-18-producing tumor cells, such as MDA-MB-231 or HeLa cells, increased the number of CD56^dim^CD16^−/dim^ NK cells and concomitantly decreased the number of CD56^dim^CD16^+^ NK cells. CD56^dim^CD16^+^ NK cells have strong antitumor activity, as these cells home to inflammatory sites and promote immune responses [15, 16]. Meanwhile, CD56^dim^CD16^−^ NK cells are more abundant at the tumor site, and express low levels of activating receptors [14]. Based on these data, tumor-derived IL-18 plays an immunosuppressive role via its regulation of NK cell subsets. Furthermore, we found that higher serum IL-18 levels in patients with EBC were significantly associated with shorter RFS and OS. Interestingly, such clinical associations were more prominent in TNBC patients, in concordance with our *in vitro* cell line data. These data suggest that the role of IL-18 may vary according to the hormone receptor and HER2 receptor status of the tumor.

Previous studies have shown that the proportion of CD56^bright^CD16^−^ NK and CD56^dim^CD16^−^ NK subsets is increased in the peripheral blood of patients with advanced breast cancer [14]. In our study, the proportions of these immature NK subsets were increased in metastatic TNBC patients. Furthermore, patients refractory to chemotherapy also manifest a higher proportion of immature NK cell subsets in their peripheral blood, and serum IL-18 levels were also elevated in these patients. Although we did not show causality between tumor derived IL-18, immunosuppressive NK cells, and resistance to chemotherapy, our data suggested a link between tumor derived IL-18 and poor responses to cytotoxic treatment. In support of these data, Yao et al. also reported *in vitro* data showing that tumor derived IL-18 contributed to the resistance to doxorubicin in breast cancer, although no correlates for NK cell subsets were provided in this analysis [17].

One of the putative metastasis-promoting roles of tumor derived IL-18 is to induce the expression of PD-1 on mature NK cells [18]. We therefore investigated PD-1 expression in each NK cell subset and the change of PD-1 expression induced by exposure to tumor derived IL-18. Upregulation of PD-1 expression was observed in CD56^dim^CD16^dim/−^ NK cells, and this upshift was suppressed following RNAi-mediated knockdown of IL-18 in tumor cells. Similar alterations in PD-1 expression were not detected in other NK subsets. Interestingly, no change in 107a and IFN-γ expression, which serve as activation markers. As a result, we assumed that tumor derived IL-18 did not affect the activities of NK cells themselves, although IL-18 increased the overall proportion of immunosuppressive NK cells. In our study, we did not find any changes in PD-L1 expression on tumor cells. This study had limitations in the investigation of the dynamic changes of tumor cells responding to various immune environment in our system.

The targeting of PD1/PD-L1 is an important therapeutic modality in cancer treatment. Anti-PD1/PD-L1 drugs have already yielded promising results in lung cancer, melanoma, and other cancers [19, 20]. In breast cancer, the clinical efficacy of this therapy was more promising in advanced TNBC, and thus, many clinical trials are focused on this specific subtype [21, 22]. PD1/PD-L1 expression can be affected by many factors in the tumor microenvironment. Understanding the mechanistic aspects of PD1/PD-L1 expression would therefore be helpful for enhancing the efficacy of these immune-modulating agents. Furthermore, tumor derived IL-18 may offer a promising target for adjunct immune therapy for cancer.

In conclusion, we show that tumor-derived IL-18 induced PD-1 expression on immunosuppressive CD56^dim^CD16^dim/−^ NK cells and is associated with poor prognosis in TNBC. Further studies are now warranted to evaluate the ramifications of tumor derived IL-18 in anti-PD1/PD-L1 treatment for breast cancer.

## MATERIALS AND METHODS

### Patients and blood samples

A total of 545 patients with operable EBC and 42 patients with triple negative MBC were recruited. For control material, peripheral blood was drawn from 24 healthy women. All patients were diagnosed with invasive breast cancer at the National Cancer Center, Korea between January 2003 and December 2010. Patients with EBC underwent surgery and then received systemic adjuvant therapy. Patients with MBC were chemotherapy-naïve or received first-line cytotoxic chemotherapy before their enrollment. For MBC patients, tumors were confirmed to be TNBC by immunohistochemistry (IHC). Blood samples were collected prior to systemic chemotherapy or surgery for patients with EBC and 6 weeks after the start of cytotoxic chemotherapy for patients with MBC. We collected clinicopathological data, including tumor size, nodal involvement, estrogen receptor (ER) status, progesterone receptor (PgR) status, HER2 status, and Ki-67 index (%) in tumor cells. For patients with MBC, we also collected data about the patients’ tumor response to chemotherapy at 6 weeks using RECIST 1.0. The NCC Institutional Review Board (IRB) approved this study (NCCNCS12657).

### Culture media, reagents, and cell lines

RPMI-1640 medium (GIBCO BRL and Invitrogen, CA, USA) was used for short-term culture of isolated human NK cells. Breast cancer cell lines were cultured in RPMI-1640 containing L-glutamine supplemented with 1% penicillin/streptomycin and 10% FBS, all of which were purchased from GIBCO BRL. IL-2 was used to culture and activate isolated NK cells, and endotoxin-free bovine serum albumin (BSA)-free recombinant human IL-18 (R&D Systems, MN, USA) was used in experiments to investigate its effects on NK cell differentiation. MCF-7, T47D, SKBR3, MDA-MB-231, and HCC-70 breast cancer cell lines as well as the IL-18-positive HeLa cell line were purchased from the Korean Cell Line Bank (KCLB, Korea). The identities of all cell lines were independently authenticated by short tandem repeat genotyping before the experiments. All cells were cultured according to KCLB recommendations.

### Evaluation of IL-18 levels in breast cancer patients and cell lines

Serum IL-18 levels were measured in culture media and cell lysates using a human IL-18 ELISA kit (Abnova, Taiwan). The detection limit was < 12.5 pg/ml. The ELISA kit was used according to the manufacturer's instructions.

### siRNA transfection of cancer cells

MDA-MB-231 and MCF7 cells were transfected with human IL-18 siRNAs (#1073737, #1073733, #1073736) or a negative control siRNA (si-NC, Bioneer, Korea), using lipofectamine RNAiMAX according to the manufacturer's instructions. After a 6-h incubation, the transfection mix was removed and replaced with complete medium. Post-transfection, cells were incubated at 37°C in a 5% CO_2_ incubator for 24–72 h.

### NK cell isolation

Peripheral blood mononuclear cells (PBMCs) from healthy donors were isolated by density gradient separation using Ficoll-HypaquePLUS (GE Healthcare, USA). NK cells were enriched by negative selection. Unwanted non-NK cells were removed using the NK cell StemSep system (StemCell Technology, Canada) according to the manufacturer's instructions. The purity and viability of sorted cells was found to exceed 90% by flow cytometry.

### *In vitro* co-culture of NK cells with breast cancer cell lines

Isolated NK cells were seeded in wells of a 12-well plate and then co-cultured with MCF-7, MDA-MB-231, or HeLa cells at ratios of 1:2 in the presence of APC-H7 anti-CD107a (BD Biosciences, NJ, USA). For siRNA transfection experiments, cells were incubated at 37°C in a humidified atmosphere containing 5% CO_2_ for 24–72 h prior to the evaluation of NK characteristics by flow cytometry.

### Flow cytometry

Co-cultured NK cells were stained using the following four monoclonal antibodies (mAb): APC- or PE-conjugated anti-CD3, PE-cy7-conjugated anti-CD16, FITC-conjugated anti-CD56, and BV510-conjugated anti-PD-1 mAbs (BD Biosciences). NK cell analyses were performed using a BD FACSVerse flow cytometer (BD Biosciences) equipped with three lasers (488 nm, 630 nm, and 405 nm). Data were analyzed using the FlowJo 10 Software (TreeStar Inc., USA).

### Immunohistochemistry

Co-cultured MDA-MB-231 cells were stained with anti-PD-L1 (BD Biosciences) and anti-IL-18 (Merck Millipore, Germany) antibodies. Cells were washed with PBS, fixed for 5 min with 4% paraformaldehyde at room temperature, washed again with PBS, permeabilized with 0.1% Triton X-100/PBS for 10 min, and then blocked with 3% normal goat serum in PBS for 10 min. Cells were then incubated at 4°C overnight with the primary antibody and then for 60 min with the secondary antibody, according to the manufacturer's instructions. Immunostaining was evaluated by fluorescence microscopy (LSM510 META, Carl Zeiss, Germany).

### Western blot

Total protein was extracted using standard protocols, and lysates were resolved via electrophoresis on SDS-PAGE gels. Separated proteins were transferred to a PVDF membrane (Bio-Rad Laboratories, Inc., CA, USA) for immunoblotting using a wet-transfer system. Following transfer, membranes were incubated overnight at 4°C with primary antibodies (diluted 1:500 or 1:1000), and bound antibody was detected using HRP-conjugated secondary antibodies (1:2000 or 1:4000; Bio-Rad Laboratories). HRP-bound antibodies were then detected on film via enhanced chemiluminescence (GE Healthcare, IL, USA).

### RNA extraction and amplification, cDNA synthesis, and RT qPCR

Total RNA was extracted from breast cancer cells using the TRIzol reagent (Invitrogen) according to the manufacturer's instructions. Then, cDNA was synthesized from 500 ng of total RNA using the SuperScript III First-Strand Synthesis System (Invitrogen). Following RT-qPCR was performed on a LightCycler 96 Real-Time PCR System (Roche Diagnostics, Germany). Data were analyzed as relative mRNA expression (quantified using the LightCycler^®^ 96 software, Roche Diagnostics) and normalized to ß-actin transcription levels.

### Statistical analyses

Comparisons between groups were made using the chi-square test for categorical variables and by the Mann-Whitney test for quantitative variables. Survival curves were constructed using the Kaplan-Meier method and compared by the log-rank test. NK cell subset proportions in the different groups were analyzed using the non-parametric Mann-Whitney *U*-test. The comparisons of marker expression between NK cell subsets were conducted using a parametric paired *t*-test. All *in vivo* experiments were graphed and calculated using the GraphPad Prism software (GraphPad Software, Inc., CA, USA). The *p*-values < 0.05 for a two-tailed test were considered statistically significant. All statistical analyses were performed using the SPSS software (SPSS Inc., IL, USA).

## SUPPLEMENTARY MATERIALS FIGURES AND TABLES


