# Supplementary material for: Tumor-derived IL-18 induces PD-1 expression on immunosuppressive NK cells in triple-negative breast cancer
Source: Oncotarget. 2017 Mar 16;8(20):32722–30. doi: 10.18632/oncotarget.16281 (PMC5464822; doi:10.18632/oncotarget.16281)
Supplement: Supplementary file 1 [file oncotarget-08-32722-s001.pdf]

Tumor-derived IL-18 induces PD-1 expression on immunosuppressive NK cells in triple-negative breast cancer

Supplementary Materials

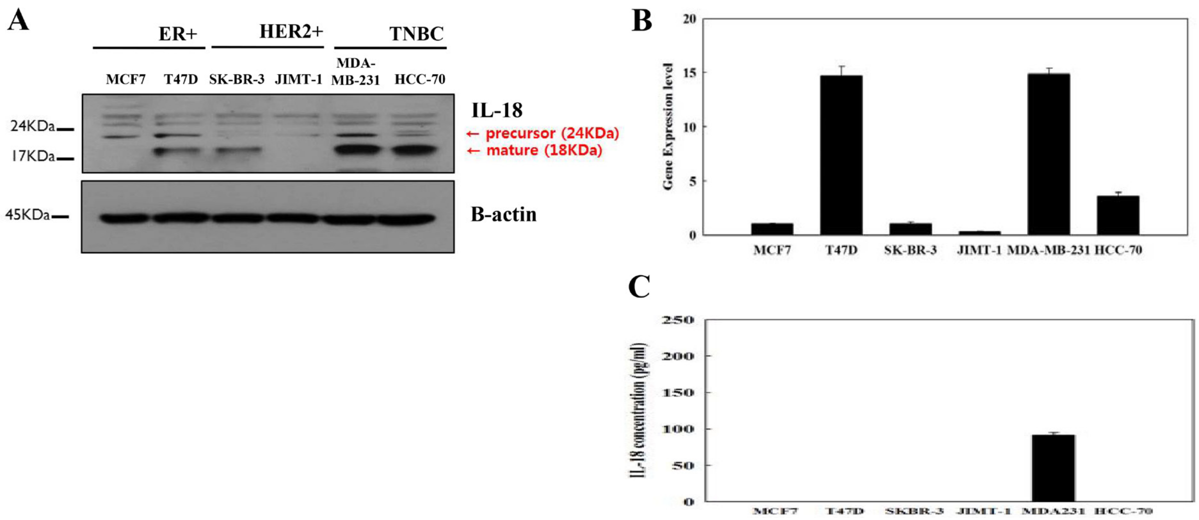

Supplementary Figure 1: IL-18 expression in breast cancer cell lines. Quantification by western blot (**A**), RT-qPCR (**B**), and ELISA (**C**). ER, estrogen receptor; TNBC, triple negative breast cancer.

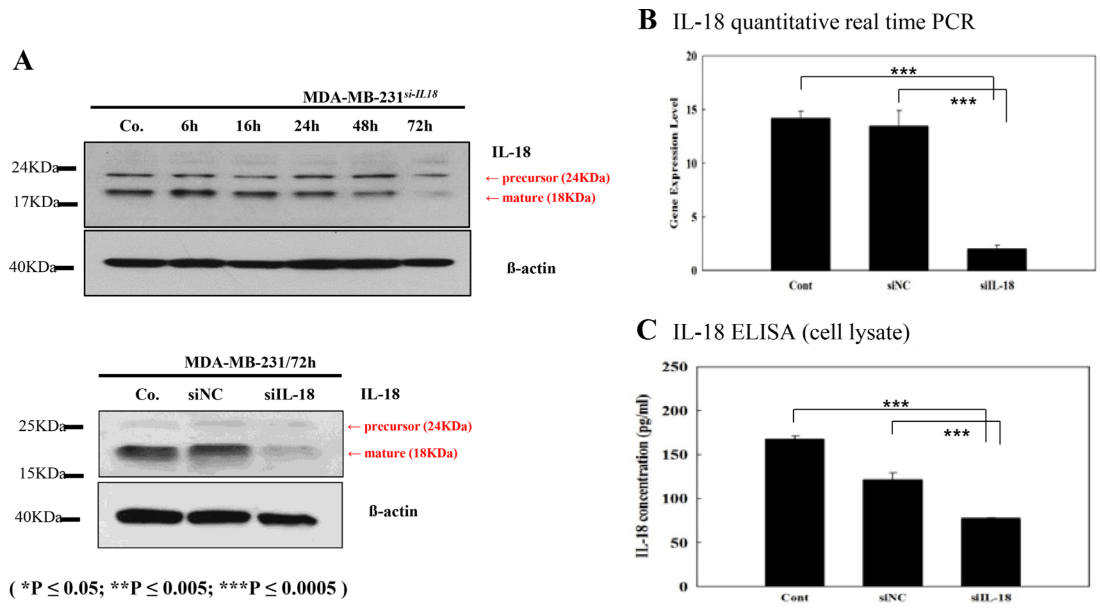

Supplementary Figure 2: siRNA knockdown of IL-18 in MDA-MB-231 cells. Western blot (**A**), RT-qPCR (**B**), and ELISA measurements (**C**) of IL-18. Bars represent normalized fold expression. C0, control not treated.

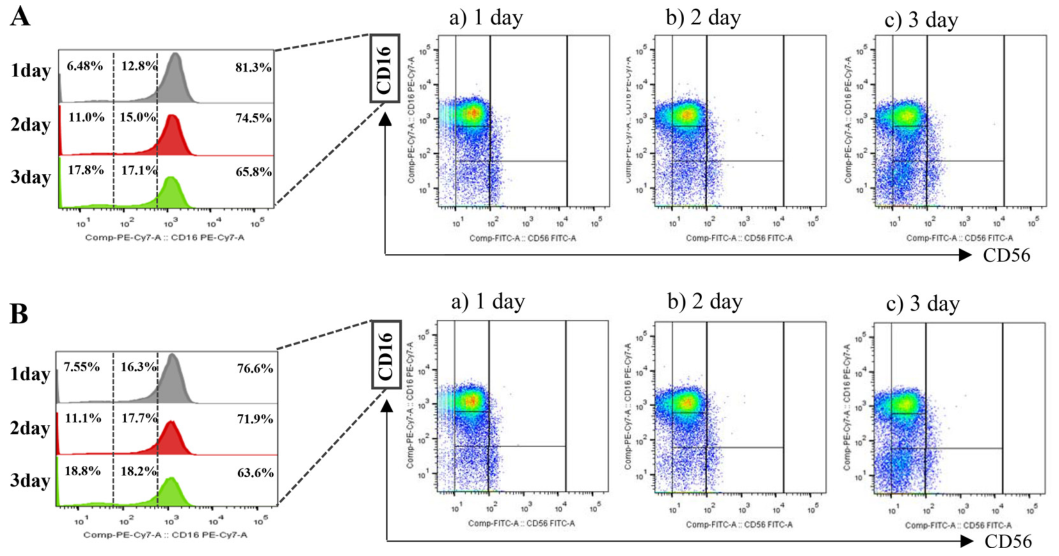

Supplementary Figure 3: Effects on the proportions of NK cell subsets after co-culture with MCF-7siNC (A) or MCF-7siIL-18 (B) cells.

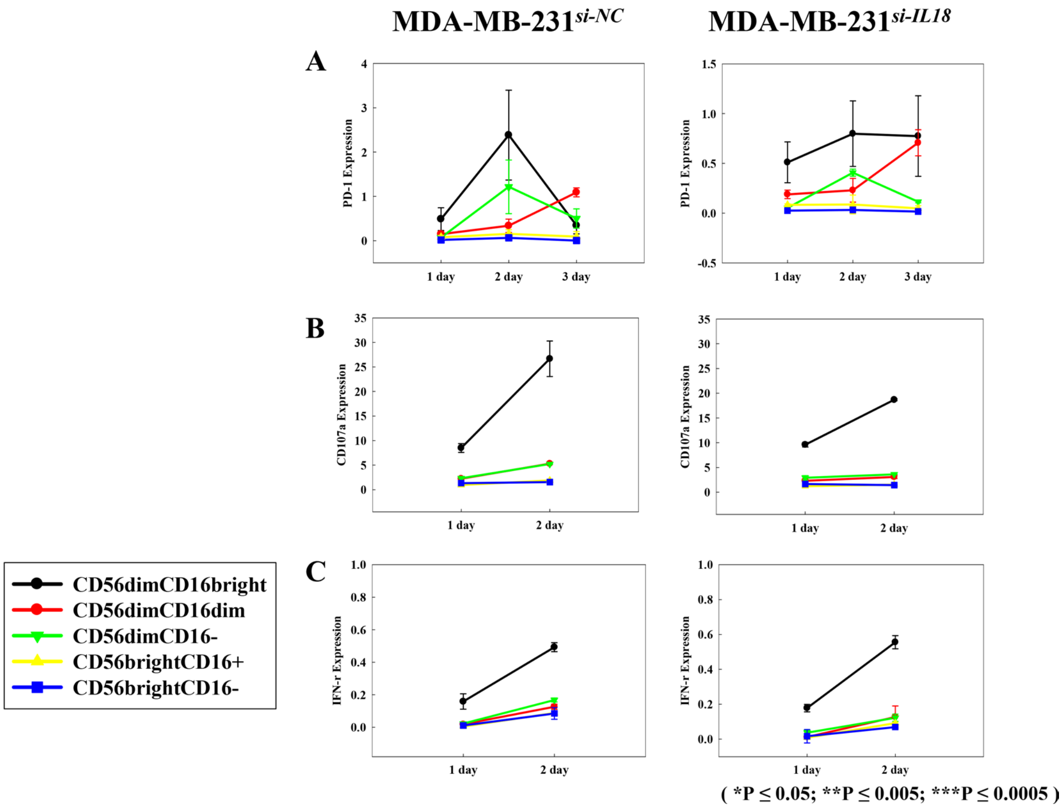

Supplementary Figure 4: The expression of PD-1 (A), 107a (B), and IFN-γ (C) on each NK cell subpopulation after co-culture with MDA-MB-231siNC or MDA-MB-231siIL-18 cells.

A

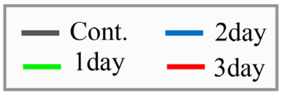

	MDA-MB-231	
PD-L1 (%)	siNC	siIL-18
1day	28.5	40.0
2day	40.8	52.4
3day	86.9	82.4

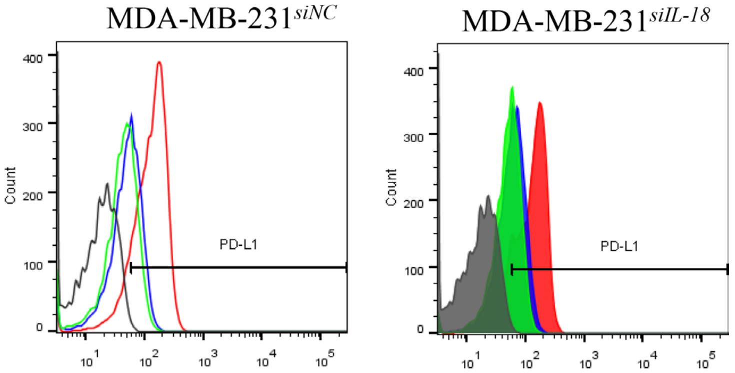

B

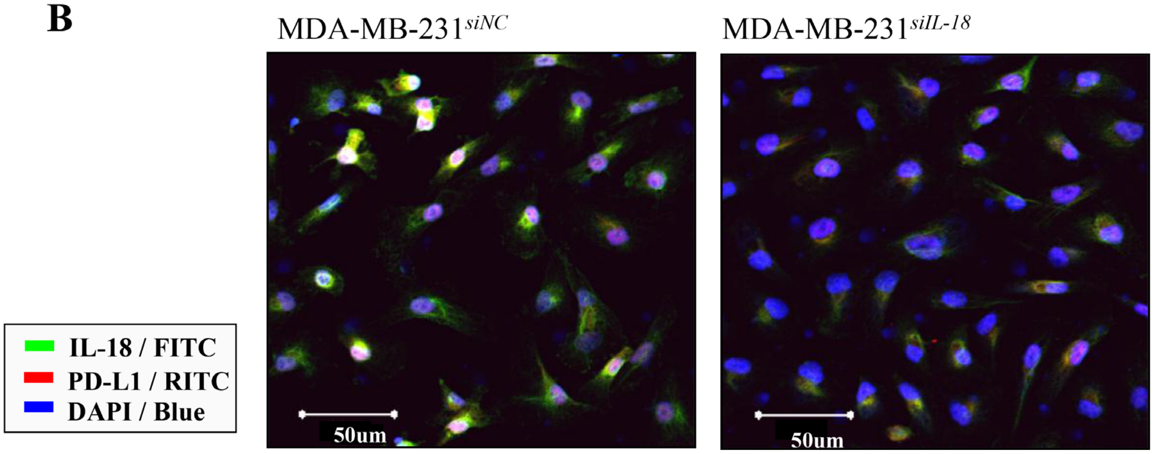

Supplementary Figure 5: PD-L1 expression on IL-18-secreting (siNC) or IL-18-depleted (siIL-18) tumor cells following co-culture with NK cells for 72 h. PD-L1 expression was analyzed by flow cytometry (**A**) or by IHC (**B**). Nuclei were stained with DAPI (blue).

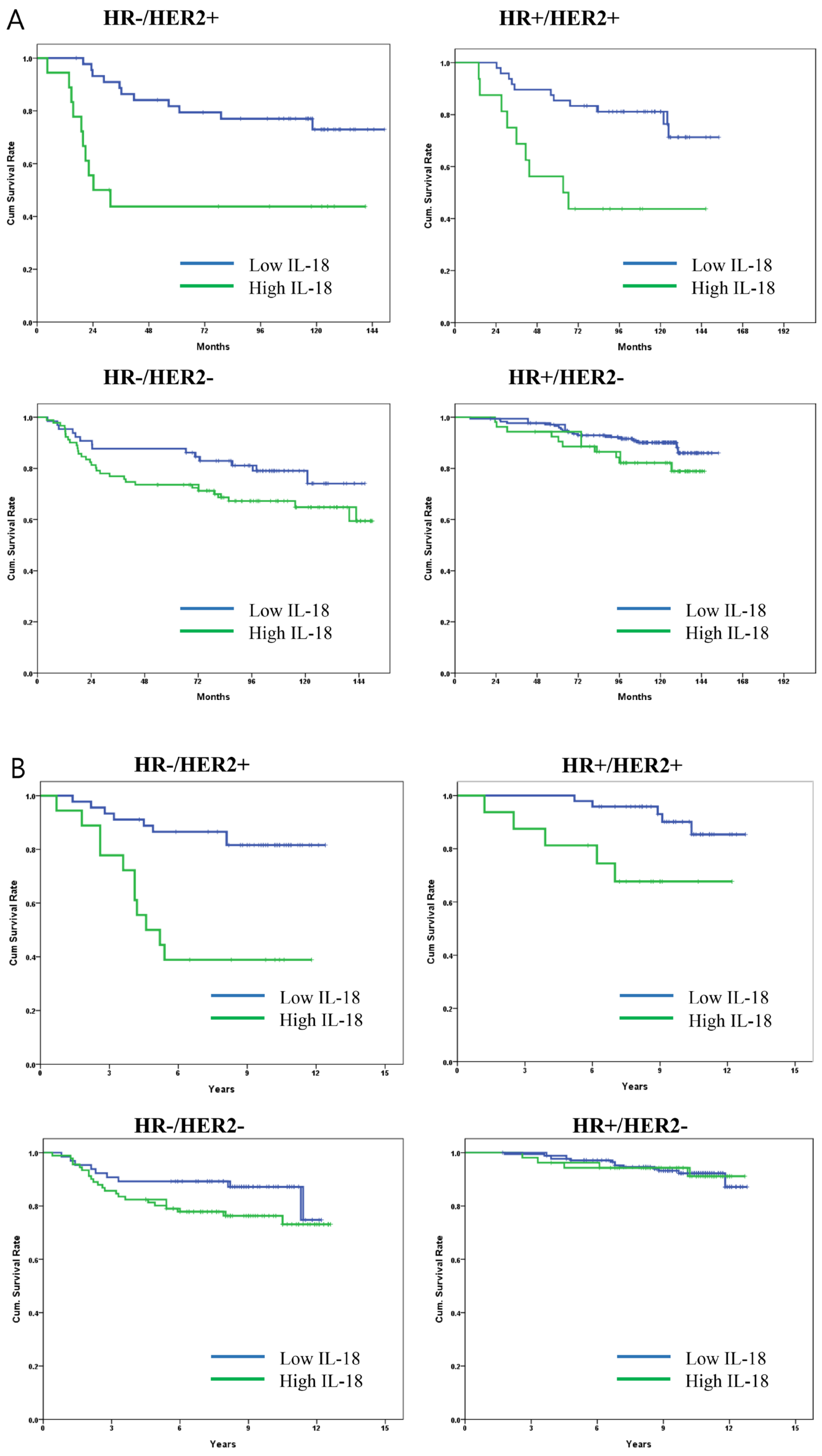

Supplementary Figure 6: Survival analyses according to serum IL-18 levels in EBC. (**A**) RFS and (**B**) OS were assessed according to HR and HER2 receptor status.

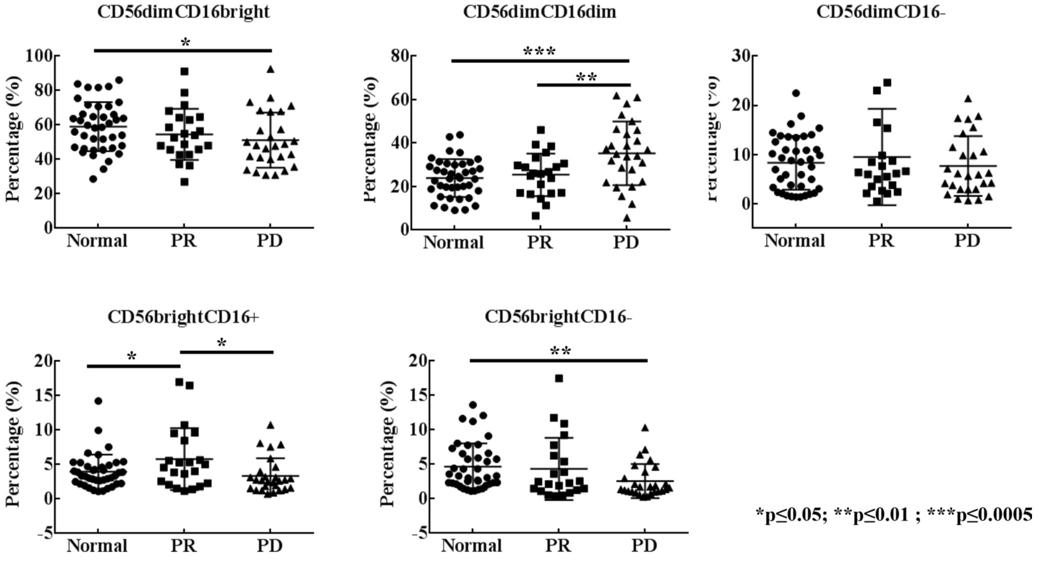

Supplementary Figure 7: NK cell subset analysis by tumor response after cytotoxic chemotherapy in metastatic TNBC patients. Control group was composed of healthy women. PR, partial response; PD, progressive disease. **p* < 0.05; ***p* < 0.001; ****p* < 0.0005.

Supplementary Table 1: Cox regression analysis for RFS and OS using various clinical factors

Variables	Univariate analysis		Multivariate analysis	
	Hazard ratio (95% CI)	*p*-value	Hazard ratio (95% CI)	*p*-value
**RFS**				
T size (> 5 cm vs. ≤ 5 cm)	5.6 (3.4–9.2)	< 0.001	3.0 (1.7–5.4)	< 0.001
N status (2 or 3 vs. 0 or 1)	3.1 (2.1–4.8)	< 0.001	1.6 (1.0–2.6)	0.052
HR (yes vs. no)	0.5 (0.4–2.8)	0.001	0.6 (0.4–0.8)	0.004
HER2 overexpression (yes vs. no)	1.9 (1.3–2.8)	0.001	1.7 (1.1–2.5)	0.016
Ki67 (high vs. low)	1.1 (0.9–1.4)	0.306	-	-
Serum IL-18 (high vs. low)	2.2 (1.5–3.2)	< 0.001	2.2 (1.5–3.2)	< 0.001
**OS**				
T size (> 5 cm vs. ≤ 5 cm)	5.7 (3.2–10.2)	< 0.001	2.8 (1.5–3.4)	0.002
N status (2 or 3 vs. 0 or 1)	4.1 (2.5–6.6)	< 0.001	2.2 (1.3–3.9)	0.004
HR (yes vs. no)	0.4 (0.3–0.6)	< 0.001	0.4 (0.3–0.7)	0.001
HER2 overexpression (yes vs. no)	1.9 (1.2–3.0)	0.006	1.5 (0.9–2.5)	0.146
Ki67 (high vs. low)	1.1 (0.8–1.4)	0.557	-	-
Serum IL-18 (high vs. low)	2.3 (1.5–3.6)	< 0.001	2.1 (1.3–3.3)	0.003

Abbreviations: RFS, recurrence free survival; OS, overall survival; CI, confidence interval; T size, tumor size; N status, nodal involvement; HR, hormone receptor; HER2, human epithelial receptor 2.

Supplementary Table 2: Characteristics of 42 metastatic TNBC patients

	PR group (*N* = 16)	PD group (*N* = 23)
Age (y, median, range)	57 (41–67)	57 (33–77)
Sex (female/male)	100%/0	100%/0
No. of previous chemotherapy (median, range)	1 (1–2)	1 (1–2)
Visceral metastasis (Y/N)	10 (62.5%)/6 (37.5%)	16 (70.6%)/7 (30.4%)
De novo stage IV disease	1 (6.2%)	2 (8.7%)

PR, partial response; PD, progressive disease.
